# Sorafenib-loaded hydroxyethyl starch-TG100-115 micelles for the treatment of liver cancer based on synergistic treatment

**DOI:** 10.1080/10717544.2019.1642418

**Published:** 2019-07-29

**Authors:** Guofei Li, Limei Zhao

**Affiliations:** Shengjing Hospital, China Medical University, Shenyang, China

**Keywords:** Liver cancer, micelles, sorafenib, TG100-115, TAMs, PI3Kγ

## Abstract

Tumor microenvironment is closely related to the occurrence and development of liver cancer. Tumor-associated macrophages (TAMs) are an important part of tumor microenvironment promoting tumor deterioration and metastasis by inhibiting immune cells. Previous studies showed that PI3Kγ inhibitor could reverse the phenotype of TAMs, relieve immunosuppression and sensitize chemotherapy drugs, suggesting that the combination of PI3Kγ inhibitor and chemotherapeutics is likely to bring new breakthroughs in the treatment of liver cancer. Based on it, this paper builds HES-TG100-115-CDM-PEG micelles with tumor microenvironment responsiveness that simultaneously loaded sorafenib and TG100-115 to synergistically treat liver cancer. Pharmacokinetic study showed that the prepared micelles had longer half-life than that of the free drug solutions, which was favorable for high propensity of extravasation through tumor vascular fenestrations. Under low pH and high α-amylasereductive conditions, micelles could depolymerize quickly due to the sensitivity of bonds and enhance significantly cytotoxic activity against Hep-3B liver cancer cell. Additionally, micelles demonstrated higher levels of antitumor efficiency and better tolerance against nude mouse with Hep-3B cell than the free drug solutions. These findings reveal that HES-TG100-115-CDM-PEG micelles are a promising drug delivery system in clinical comprehensive therapy of liver cancer.

## Introduction

Liver cancer is a common malignant tumor and has become a major disease threatening human health (Liu et al., 2015). The number of newly diagnosed liver cancer patients in China account for 60% of the world’s total, and the mortality rate is the second highest in malignant tumors. Although the proliferation, invasion, and metastasis of cancer cells are the main causes of death of patients with liver cancer, large numbers of stromal cells (about 90% non-cancer cells) in tumor microenvironment are the key drivers of the progress of liver cancer (Wu & Dai, 2017; Tahmasebi & Carloni, 2017). Therefore, the treatment of liver cancer should not be limited to killing tumor cells, and remodeling the tumor microenvironment may become an important breakthrough for liver cancer treatment.

Tumor-associated Macrophages (TAMs) are important components of tumor microenvironment and play an important role in tumor progression. Studies on patients with liver cancer have shown that a large number of M2-type TAMs infiltrate at the edge of blood vessels, which can inhibit immunity and promote tumor angiogenesis, matrix remodeling and metastasis of cancer cells (Degroote et al., 2018). As the multiple roles of TAMs are gradually revealed, lots of studies have been invested in tumor immunotherapy based on targeted TAMs, and a series of breakthroughs have been made in regulating the phenotype of TAMs and relieving tumor immunosuppression (Sun, 2016). Therefore, how to regulate TAMs phenotype to reshape tumor microenvironment may be the key to effectively inhibit liver cancer.

The phosphoinositide-3-kinase gamma (PI3Kγ) pathway is one of the hotspots in the study of TAMs. PI3Kγ inhibitors can remodel the tumor microenvironment by reversing TAMs phenotype (Kaneda et al., 2016). Literature shows that: PI3Kγ inhibitors can change the balance of immunosuppressive cells such as TAMs and promote the activation of anti-tumor immunity (Amano et al., 2018). Meanwhile, PI3Kγ inhibitors can improve the responsiveness of TAMs-enriched tumors to chemotherapy drugs (cisplatin, etoposide, and fluorouracil) and checkpoint blocking therapy (Wang et al., 2018). For example, the efficiency of checkpoint inhibitor alone in the treatment of immune-tolerant mice was only 20%, while it reached 80% when combined with TG100-115 (Hsu et al., 2018; Wang et al., 2018). This suggests that chemotherapy combined with immunotherapy may be a new tumor treatment mode superior to any single therapy based on the crosstalk between tumor cells and microenvironment.

Sorafenib is a tyrosine kinase inhibitor and a first-line drug for the treatment of advanced liver cancer (Yang et al., 2017; Huang et al., 2018). Although sorafenib can inhibit the growth of tumors in situ, it mobilizes a large number of macrophages into the blood and promotes the activation of macrophages to M2 type, resulting in great trouble for the treatment and prognosis of liver cancer (Wei et al., 2017). While TG100-115 is an exclusive PI3Kγ inhibitor, which can improve the progression of various types of tumors (including liver cancer, lymphoma and breast cancer) by reversing the phenotype of TAMs, inhibiting tumor angiogenesis, enhancing the recruitment of cytotoxic T lymphocytes, and relieving immunosuppression (Setti et al., 2016). Thus, the combination of sorafenib and TG100-115 may produce significant synergistic effects and improve antitumor efficacy.

In recent years, polymer-drug conjugate micelles have provided a new weapon for the synergistic treatment of liver cancer based on tumor microenvironment regulation and chemotherapy (Shi et al., 2017; Zhao et al., 2017; Li et al., 2018; Zhang et al., 2018). Polymer-drug conjugate micelles possess several obvious advantages over free drugs (Feng & Tong, 2016; Abdelmoneem et al., 2018; Hanafy et al., 2018): (1) co-loaded different types of drugs by chemical bonding and physical package; (2) improve the solubility of poorly soluble drugs and control the release behavior of drugs; (3) prolonged *in vivo* circulation time by escaping the uptake of the reticuloendothelial system; (4) selective distribution of micelles in tumor tissue by utilizing the EPR effect of tumor blood vessels. Additionally, it is well known that abnormal energy metabolism of tumor cells leaded to low pH, over-expressed enzymes and glutathione of tumor tissues, which can be used as targets for targeting tumor microenvironment to enhance the selectivity of nanomedicines, and many research achievements have been made in the diagnosis and treatment of tumors. Thus, it is necessary to endow nanomedicines with tumor microenvironment responsiveness to deliver drugs selectively to tumor cells and TAMs based on the specificity of tumor microenvironment and the spatial distribution characteristics of different subcell groups.

Although lots of polymers have been used to prepare polymer micelles, only a few have been successfully used in clinical practice. Hydroxyethyl starch (HES) and polyethylene glycol (PEG) are nontoxic, nonimmunogenic polymer with excellent biocompatibility and hydrophilicity (Hu et al., 2016; Li et al., 2016). Meanwhile, they all have excellent modification, long circulation effects in the body, well self-assembly performance in aqueous environment, and strong drug-loading ability, which make HES and PEG received extensive attention as nanomedicine carrier. Thus, the purpose of this study was to construct HES-TG100-115-CDM-PEG polymer micelles with tumor microenvironment sensitivity and co-loaded sorafenib for the treatment of liver cancer. The versatility of the prepared micelles can improve the effective accumulation of the drugs in the tumor tissue, reduce systemic toxicity, and improve tumor microenvironment, thereby increasing therapeutic effect.

## Materials and methods

### Material and reagents

HES (130 kDa/0.4) were obtained from Chongqing Daxin Pharmaceutical Co., Ltd. (Chongqing, China). 2-Propionic-3-methyl maleic anhydride and PEG 5000 were obtained from Aladdin Shanghai Co., Ltd. (Shanghai China). TG100-115 and sorafenib were purchased from Shanghai Biochepartner Co., Ltd. (Shanghai, China). MTT was furnished from Sigma. All chemicals and reagents used were of analytical or chromatographic quality.

### Synthesis of HES-TG100-115-PEG

HES-CHO was synthesized based on the oxidation reaction reported by Maria Kavallaris et al. Briefly, sodium periodate (NaIO4; 4.3 g, 0.020 mol) was dissolved in deionized water (100 mL). The solution was protected from light. After adding HES (3.0 g, Mw 130000 g/mol) in the sodium periodate solution, the mixture was stirred overnight at room temperature in the dark. After the reaction was completed, the product was extensively dialyzed against water to remove excess reactant using cellulose membrane (MWCO of 3500 g/mol). The purified aldehyde functionalized HES was lyophilized to obtain a white powder (5.1 g). HES-TG100-115 was synthesized through imine bond reaction. Typically, 1.5 g of HES-CHO and 0.2 g of TG100-115 were mixed in a DMSO solution and stirred at 40 °C for 48 h. The unreacted TG100-115 was removed through dialysis (MWCO of 3500 g/mol) against 20% DMSO-deionized water solution for 48 h. After filtration, the solutions were slowly added to ice-ethanol and stirred constantly to produce precipitate and the precipitates were dried under vacuum at 40 °C for 24 h to obtain final HES-TG100-115 conjugates. 120 mg 2-Propionic-3-methyl maleic anhydride was dissolved in 10 mL dichloromethane, and the solution was mixed with 100 mg oxalyl chloride and 150 µL DMF. The solutions were stirred for 10 min on ice, then 1 h at room temperature. After vacuum drying, residual chlorine-substituted CDM was dissolved in dichloromethane, 200 mg of PEG was added, and the mixtures were stirred for 8 h at room temperature. The mixtures were precipitated in cold ethyl ether. The products were filtered and dried overnight in vacuum, and PEG-CDM was obtained as a light brown solid with 70% yield. PEG-CDM-HES-TG100-115 was synthesized by a ring-opening reaction of PEG-CDM with HES-TG100-115. Briefly, 200 mg HES-TG100-115, 50 mg PEG-CDM and catalyst were mixed in 15 mL DMSO solution and stirred at 45 °C. After 12 h, the solutions were slowly added to ice-ethanol and stirred constantly to produce precipitate and the precipitates were dried at 40 °C for 24 h to obtain PEG-CDM-HES-TG100-115 conjugates. The NMR technique was used to verify the structure of products.

### Preparation of HES-TG100-115-CDM-PEG micelles

Appropriate amounts of HES-TG100-115-CDM-PEG and sorafenib were dissolved in 5.0 mL of DMSO organic solvent. After completely dissolved, the mixed solutions were poured into dialysis bag and dialyzed against 1.5 L of deionized water for 48 h. The dialysis turbid solution was centrifuged at 1000 rpm for 10 min, and the supernatant was filtered through a 0.45 μm filter membrane to remove the unencapsulated sorafenib. Finally, the filtrate was freeze-dried to obtain the HES-TG100-115-CDM-PEG micelles loaded sorafenib. The properties of the prepared micelles were characterized by dynamic light scattering and transmission electron microscopy, including particle size distribution, surface morphology, dispersion state, etc.

### *In vitro* release of TG100-115 and sorafenib

#### Release of the drugs from micelles in PBS solution

Briefly, micelles solution with a concentration of 0.5 mg/mL was prepared using phosphate buffer saline (PBS) solution. Then, place the dialysis bag (MWCO of 3500 g/mol) containing the above micelles solution in 35.0 mL fresh PBS solution to investigate the drug release behavior. The pH value of PBS solutions was 5.5, 6.8 and 7.4 respectively. Take 0.5 mL of dialysate at regular intervals, determine the concentration of the drugs by HPLC method, and add 0.5 mL fresh PBS solution.

#### Release of the drugs from micelles in plasma and liver homogenate solution

Forty percent rat plasma and liver homogenate were obtained by dilution with physiological saline for drug release studies. Then, 10.0 mg micelles were dissolved in 15 mL above plasma and liver homogenate respectively. The release conditions are the same as in PBS solution. At pre-determined intervals, 0.2 mL samples were taken for determination of drug concentrations using HPLC method.

### Cytotoxicity assay

The cytotoxicity test of HES-TG100-115-CDM-PEG micelles loaded sorafenib against human liver cancer cell lines Hep-3B was studied by MTT assay. The liver cancer cell line Hep-3B was seeded into 96-well plates at a density of 6 × 10^3^ cells/well. Cells were incubated in an incubator under 5% CO_2_ at 37 °C for 24 h and, subsequently, the medium was removed, and the micelles in different concentrations were added to the cell wells for 48 h and 72 h. Positive control treatments included different concentrations of free sorafenib and TG100-115, and a negative control with medium alone. Then, 50 μL MTT (0.25 mg/mL) was added to each well at predetermined times. After 4 h incubation period at 37 °C, the supernatant was removed, and 100 μL DMSO was added to dissolve the precipitated formazan crystals. The plates were shaken for 10 min and the OD values were measured with an ELISA reader at a wavelength of 492 nm. The apparent cytotoxicity was calculated according to the following equation, and the concentrations of the test agent producing a 50% reduction in cell numbers were compared with control cultures.

Cell inhibition ratio (%)=1 − OD value of sample/OD value of negative control × 100%

### Pharmacokinetics of the prepared micelles

Sprague-Dawley rats were randomly divided into three groups and each group contains 6 rats. The prepared micelles solution was injected through femoral vein and the dose was 20 mg/kg (sorafenib) and 30 mg/kg (TG100-115). Free sorafenib and TG100-115 solutions were used as reference and the dose is same as the micelles group. After the end of administration, appropriate blood was obtained from the retro-orbital sinus at preselected time intervals (5, 10, 15, 30, 60 min, 2, 4, 6, 8, 12, 24, 48 and 72 h). Then, the blood was centrifuged at 3500 rpm for 10 min to acquire final plasma.

The plasma concentration of sorafenib and TG100-115 was measured using a validated HPLC-MS/MS method. The specific conditions are as follows: poroshell 120 poroshell-C_18_ column (50 mm × 4.6 mm, 2.7 μm, Agilent) was chosen as an analytical column, and the column temperature was 40 °C. The mobile phase comprised acetonitrile and 10 mmol/L ammonium acetate containing 0.1% formic acid, and the flow rate was 0.7 mL/min. Imatinib and naproxen were chosen as internal standard (IS) to improve the accuracy of the measurement. The ion ionization of sorafenib and imatinib was positive ion ionization, while TG100-115 and naproxen were negative ion ionization. The specific MS parameters of analytes were shown in S1 (Supplemental 1).

Plasma samples are processed according to the standard operating practices described below. Briefly, add 150.0 μL of plasma sample and 20.0 μL of the IS solution in a 2.0 mL EP tube. Then, the solution was vortexed for 1.0 min and added 300 μL of ice acetonitrile to precipitate protein. After vortexing for 1.0 min, the solution was centrifuged at 15000 rpm for 10 min. After that, 150 μL of supernatant was collected for quantitative analysis.

### *In vivo* pharmacodynamic evaluations

The *in vivo* anti-tumor activity and safety of the prepared micelles was investigated by using Hep-3B-xenografted nude mouse model. The mice were randomly divided into six groups (*n* = 6) when the tumor volumes reached around 150 mm^3^. The tumor-bearing mice were treated with non-targeted micelles solution, targeted micelle solution, TG100-115 solution, sorafenib solution, mixed sorafenib/TG100-115 solution and normal saline. The dose of sorafenib and TG100-115 was 20 and 30 mg/kg respectively. The injection was given twice a week for 4 weeks and the tumor volume/body weight was measured every 2 days. The mice were sacrificed on day 28. Thereafter, the tumors were totally excised and precisely weighed. The tumor inhibitory rates were calculated using the formula: Tumor inhibition rate (%) = (V_blank _−V_sample_)/V_blank_ × 100%, V_blank_, and V_sample_ represent the tumor volumes in the blank and test, reference group respectively.

### Evaluation of micelles renal toxicity

It is well known that high molecular polymers are excellent carriers for the delivery of drugs, but their potential toxicity should cause us enough attention, especially kidney toxicity. Thus, the kidney toxicity of HES-CDM-PEG was evaluated by administrating micelles solutions to rats. The concentration of HES-CDM-PEG is 100 mg/mL and 500 mg/mL respectively. The frequency of administration is once a day, and the administration period is 3 weeks. The changes in kidney tissue were observed by kidney tissue imaging.

## Results and discussion

### Synthesis and characterization of the prepared micelles

The final products are confirmed by ^1^H NMR mainly, and the results are shown in S3. HES-CHO: the following conclusions are obtained by comparing the ^1^H NMR spectrum of HES-CHO with the related literature due to the ^1^H NMR spectrum of HES has a serious overlap. When the chemical shift value is in the range of 5.6–4.0 ppm, it should be H on the C1 position and the hydroxyl group of the sugar ring. A characteristic peak of the aldehyde group appeared on the spectrum after the formation of HES-CHO, and the chemical shift value was about 8.0 ppm. Thus, the synthesis of HES-CHO was successful and the entire synthesis process showed good reproducibility and controllability. HES-TG100-115 conjugate was prepared by imine bond between HES and TG100-115. The characteristic resonance of imine bond can be seen at 7.6 ppm. Additionally, it can be found the characteristic resonance of TG100-115 on the ^1^H NMR spectrum, concluding single peak (at 7.150 ppm), and multiple peaks (at 6.75, 6.81, 6.88, 6.91 ppm). CDM is introduced into PEG by the reaction of carboxyl group and hydroxyl group. The characteristic resonance of methylene of CDM can be seen at 2.75 and 3.26 ppm. The resonance of methylene of PEG is at 3.7 ppm. In addition, the presence of ester bond causes significant change in the chemical shift value of the characteristic peak of PEG. HES-TG100-115-CDM-HES was obtained by the reaction of carboxyl group of CDM and hydroxyl group of HES. In addition to the ^1^H NMR used to confirm the structure of HES-TG100-115-CDM-HES, TG100-115 has two different forms pH 7.4 PBS solution due to the formation of conjugates, free TG100-115, and the conjugate respectively. Two different forms of TG100-115 present different peak positions on the HPLC chromatogram because of polarity differences, and the retention time were 5.7 and 11.4 min, respectively. Here, the retention time of HES-TG100-115 conjugate was 5.7 min. Thus, the synthesis of HES-TG100-115-CDM-HES conjugate is successful.

### Preparation of HES-TG100-115-CDM-HES micelles

The HES-TG100-115-CDM-PEG conjugates showed amphiphilic properties with PEG and HES act as hydrophilic parts and TG100-115 as hydrophobic moiety. Thus, HES-TG100-115-CDM-PEG conjugates can self-assemble easily into polymer micelles in aqueous environment due to the presence of hydrophobic force. Here, the hydrophilic shell is made up of HES; TG100-115 constitutes the core of the micelles, while PEG is attached to the surface of HES in the form of a crown to escape the phagocytosis of the reticuloendothelial system. The critical micelles concentration (CMC) of HES-TG100-115-CDM-PEG micelles is detected to be 9.8 μg/mL. The surface morphology of the prepared micelles was observed by transmission electron microscopy, and the spherical structure was shown in S4. Additionally, the particle size distribution of the micelles measured by dynamic light scattering was 71.3 ± 8.9 nm. It is well known that pharmacokinetics and pharmacodynamics are closely related to the drug loading of the micelles. In this study, the hydrophobic sorafenib is physically contained in the core of the micelles in the process of self-assembly, while TG100-115 was loaded by chemical conjugate. Chemically bonded drug-loading methods have obvious advantages compared with physically drug-loading method. On the one hand, the stability of the drug during circulation was improved by constructing environmentally sensitive bonds (an imine bond), and the early release of the drug was avoided effectively. On the other hand, the drug loading can be well adjusted according to the needs of the study. In this study, sorafenib can be stably contained in the core of micelles due to the presence of the hydrogen bonding between sorafenib and TG100-115 and HES.

### *In vitro* drug release of the micelles

The *in vitro* drug release behaviors of micelles were evaluated in PBS solutions (pH 5.5, 6.8, 7.4), plasma and liver homogenate at a concentration of 40% using the dialysis method, and the specific results were shown in S5.

It was found that the amount of TG100-115 and sorafenib released from HES-TG100-115-CDM-PEG micelles was 19% and 28%, respectively in 72 h at pH 7.4 PBS solutions. This is mainly due to the fact that the structure of the micelles remains intact under the condition of pH 7.4, which guarantees the selective accumulation and good blood circulation stability of micelles. When the pH of PBS was 6.8, the release of TG100-115 and sorafenib increased, reaching 45% and 60% in 72 h, respectively. That is because the bond (amide bond and CDM bond) can break rapidly under conditions of pH 6.8, especially the CDM bond. The breakage of the bonds causes depolymerization of the micelles, allowing the encapsulated sorafenib and chemically linked TG100-115 to be released quickly. Similar results can be obtained at pH 5.5 PBS solutions, the release of TG100-115 and sorafenib reached 52% and 65% in 72 h, respectively. In summary, the release of the drug in the micelles shows a significant selectivity because of the pH responsiveness of the linkage, which ensures that the micelles can release drugs quickly in tumors and cells to achieve therapeutic effects. However, TG100-115 and sorafenib cannot release completely in pH 6.8 and 5.5 PBS solutions, which may be related to the stereoscopic protection effect of HES, delaying the breakage of the bond and the depolymerization of the micelles. Here, 10 mM α-amylase was added to the release medium respectively in order to investigate the effects on drug release. It was found that α-amylase can significantly increase the release of the drug in comparison with above PBS solutions. The release of sorafenib in PBS solution (pH 5.5, 6.8, 7.4) was 33%, 65% and 75% in 72 h, respectively. The release of TG100-115 in PBS solution (pH 5.5, 6.8, 7.4) was 23%, 51% and 62% in 72 h, respectively. It is well known that HES can be degraded into low molecular weight starch units under the action of amylase by breaking the α-1,4 glycosidic bond, resulting in destruction of the micelle structure and rapid release of the drug. It can be concluded that the sorafenib and TG100-115 can be released to the maximum extent in the tumor tissue because tumor tissue had lower pH, high concentration of α-amylase and other hydrolases compared with normal organization. To further investigate the stability of micelles, plasma and liver homogenates were selected to evaluate the release behavior of micelles. It was found that the release quantity of TG100-115 and sorafenib from the micelles in rat plasma was 23% and 30% in 72 h respectively. That is to say, there is no significant difference in the release behavior of the drug in plasma and pH 7.4 PBS solutions. The reason may be as following: the linkage (amide bond and CDM bond) is pH sensitive and is stable in plasma (pH7.4); Diluted plasma (40%) reduces the degradation of HES by the α-amylase, allowing the stereoprotective effect of HES to be exerted. The presence of PEG further reduces the effect of α-amylase and pH on the micelles and improves the stability of the micelles. In contrast, the release quantity of TG100-115 and sorafenib from the micelles in liver homogenate was 44% and 55% in 72 h, which were higher than in rat plasma. This may be due to the presence of more enzymes in liver homogenate, which accelerated the destruction of micelles structure, breaking of the connection bonds and decrease of the stereo-protective effect of HES. Sum up, the prepared micelles exhibited apparent blood circulation stability and intelligent release of drugs, which will contribute to the delivery of micelles and the efficacy of the drugs.

### *In vitro* cytotoxicity test

**Figure 1.**
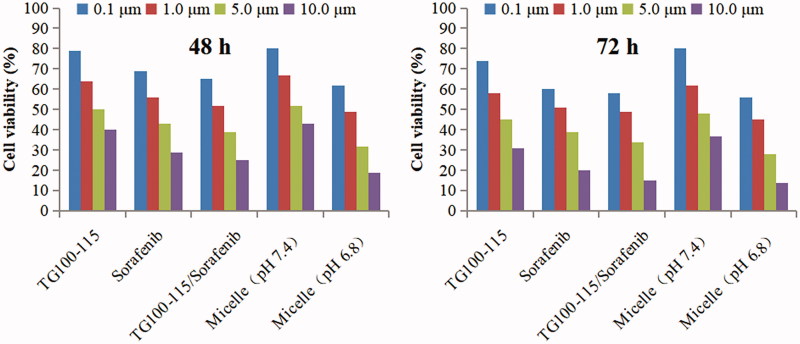
Results of MTT assay on Hep-3B cells after incubation of 48 and 72 h with drugs solutions and micelles at various concentrations.

The effect of HES-CHO and the prepared micelles on the proliferation of the Hep-3B cells are investigated using MTT assay, and the results are shown in Figures 1 and S2.

The following conclusions can be obtained from the MTT assay results: (1) HES-CHO showed good safety and biocompatibility, with no significant inhibition effects on Hep-3B cells at all concentrations (5.0, 25.0, 100.0, 250.0 mg/mL). (2) Cytotoxicity was positively correlated with the dose. The free drugs solution showed greater cytotoxicity than the micelles incubated at pH 7.4. This mainly due to the micelle-loaded drugs can not directly act on the cells because the release of drugs in micelles is slow at pH 7.4. Thus, the micelles incubated at pH 7.4 showed lower cytotoxicity than the drugs solution. In contrast, the micelles incubated at pH 6.8 showed higher cytotoxicity than the micelles incubated at pH 7.4. That is because the bond can break rapidly under conditions of pH 6.8, which cause depolymerization of the micelles and release of the encapsulated sorafenib and chemically linked TG100-115. Therefore, the micelles incubated at pH 6.8 showed similar cytotoxicity with the free drugs solution. The results of the MTT test further confirmed that the micelles can release the drug smoothly and exert anti-tumor effect in the special microenvironment of tumor tissue.

### Pharmacokinetics of HES-TG100-115-PEG micelles

**Figure 2.**
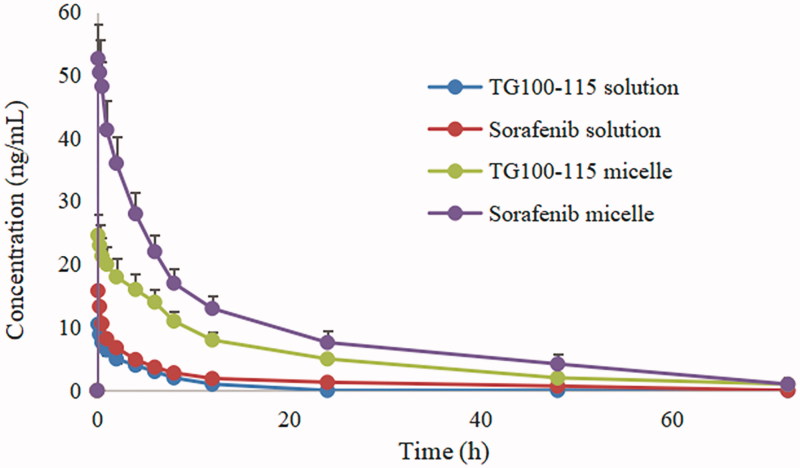
The rat plasma concentration versus time curves of micelles and drug solution after intravenous administration.

Long circulation *in vivo* is the most basic feature of a qualified nanomedicine. Only with long circulating properties, nanoparticles can enter the tumor tissue through the EPR effect of tumor blood vessels, and exert the efficacy of the drugs. Therefore, how to effectively prolong blood circulation time of nanomedicines and avoid the phagocytosis of the reticuloendothelial system is an important guarantee to increase the accumulation and distribution of drugs in tumor tissue. The mean plasma concentration-time curves and corresponding pharmacokinetic parameters are presented in Figure 2 and Table 1 respectively.

**Table 1.** Pharmacokinetic parameters of TG100-115 and sorafenib after intravenous administration of different formulations (mean ± SD).

Pharmacokinetic parameters	TG100-115 solutions	Sorafenib solutions	TG100-115 micelles	Sorafenib micelles
AUC_(0-t)_ (μg/L·h)	43.4 ± 10.2	74.5 ± 18.7	2278.6 ± 432.1^a^	3429.6 ± 643.7^b^
AUC_(0-∞)_ (μg/L·h)	45.8 ± 11.7	78.6 ± 20.6	2301.5 ± 441.4^a^	3502.1 ± 654.8^b^
CL_z_ (L/h/kg)	32.7 ± 5.5	25.4 ± 3.7	5.6 ± 1.5^a^	2.5 ± 0.43^b^
C_max_ (μg/L)	9.5 ± 2.0	17.3 ± 3.2	25.6 ± 4.7^a^	51.5 ± 8.6^b^
*t_1/2_* (h)	1.7 ± 0.3	8.8 ± 1.4	14.5 ± 3.2^a^	36.7 ± 7.3^b^
T_max_ (h)	0.167 ± 0.045	0.167 ± 0.073	0.50 ± 0.12	0.167 ± 0.088

^a^*p* < .05 compared to TG100-115 solutions group.

^b^*p* < .05 compared to sorafenib solutions group.

It can be seen that the half-life of free TG100-115 and sorafenib solution was 1.7 h and 8.8 h respectively from Table 1. The short half-life of TG100-115 may limit its efficacy to some extent, especially as a channel inhibitor. In comparison, sorafenib shows a longer half-life, but it has significant systemic toxicity due to lack of tissue selectivity. The half-life of TG100-115 and sorafenib was extended to 14.5 h and 36.7 h respectively after making HES-TG100-115-CDM-PEG micelles. That is to say, the circulation time of the drugs *in vivo* has been obviously improved after the formation of micelles, especially for TG100-115.

The reasons why HES-TG100-115-CDM-PEG micelles have good long-term circulation effects *in vivo* may be as follows: (1) the hydrophobic interaction of the conjugate and the hydrogen bonding between HES-CHO and sorafenib significantly increase the stability of the micelles. In addition, HES can only be slowly degraded by the α-amylase. Thus, the release rate of the drugs is significantly limited by above forces. (2) the release of TG100-115 was carried out under acid conditions because amide bond between TG100-115 and HES is acid sensitive, which results in a significant sustained release effect under physical conditions. Meanwhile, the good physical stability of the micelles also delays the release of sorafenib to some extent. All of these properties made HES-TG100-115-CDM-PEG micelles showed good stability and obvious long circulating effect.

### Pharmacodynamics of HES-TG100-115-CDM-PEG micelles

**Figure 3.**
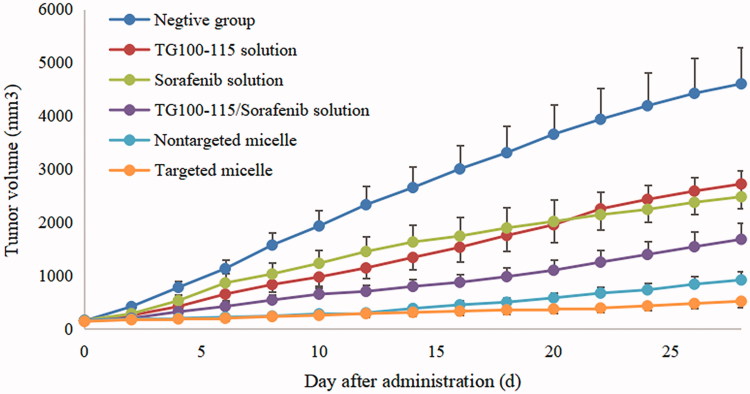
The tumor growth curve of nude mouse after intravenous administration of micelles and drug solutions.

*In vivo* pharmacodynamics of micelles was evaluated in nude mouse bearing Hep-3B cells, and the specific results are presented in Table 2 and Figure 3.

**Table 2.** Anti-tumor effects of TG100-115 and sorafenib preparations against Hep-3B cell in nude mouse.

	Dose (mg/kg)	Body weight (g)		Tumor weight (g)	IRT (%)
		Initial	Final		
Normal saline	–	23.4	38.6	4.60 ± 0.52	–
TG100-115 solution	30	22.7	35.7	2.72 ± 0.31	40.87
Sorafenib solution	20	23.6	34.2	2.48 ± 0.36	46.09
TG100-115/sorafenib solution	30/20	23.1	34.9	1.68 ± 0.22	63.48^a,^^b^
Non-targeted micelle	30/20	22.5	33.3	1.18 ± 0.19	74.35^a,^^b,^^c^
Targeted micelle	30/20	24.2	33.8	0.52 ± 0.10	88.70^a,^^b,^^c,^^d^

^a^*p* < .05, compared with TG100-115 solution group.

^b^*p* < .05, compared with sorafenib solution group.

^c^*p* < .05, compared with TG100-115/sorafenib solution group.

^d^*p* < .05, compared with Non-targeted micelle group.

It was found that the growth rate and extent of tumors in the negative control group were much higher than other experimental groups, and the tumor volumes reached appropriate 4600 mm^3^ at the end of the test. Compared to the negative control group, TG100-115 and sorafenib solution groups shown a certain anti-tumor efficacy. The average tumor weight of the free TG100-115, sorafenib was 2.72 ± 0.31 and 2.48 ± 0.36, and the tumor inhibition rate reached 40.87% and 46.09% respectively. It can be seen that both free TG100-115 and sorafenib solutions showed inhibition effect of tumor, but not enough to significantly inhibit the progression of the tumor. In the case of TG100-115, the efficacy is limited significantly because it is difficult to maintain therapeutic effect for a long time due to its relatively short half-life, which reduced drug efficacy. For sorafenib, it has dual anti-tumor effect according to the reports (Dong et al., 2019). It can directly inhibit the proliferation of tumor cells by blocking the RAF/MEK/ERK-mediated cell signaling pathway, and can also inhibit tumor growth by inhibiting the formation of new blood vessels by acting on VEGFR (Hage et al., 2019). However, although sorafenib can inhibit the growth of tumors *in situ*, but it can mobilize a large number of macrophages into the blood and promotes the activation of macrophages to M2 type. While M2 type TAMs can express a variety of anti-inflammatory cytokines and cause great trouble for the treatment and prognosis of liver cancer, resulting in enhanced invasion and metastasis of liver cancer cells. Thus, the antitumor effects of TG100-115 and sorafenib alone have been significantly inhibited. Given the different antitumor mechanisms of TG100-115 and sorafenib, the combination of TG100-115 and sorafenib may improve the antitumor activity due to the presence of synergistic anti-tumor effect between TG100-115 and sorafenib. TG100-115 as a new generation of PI3Kγ inhibitors, which can activate adaptive immunity, enhance the recruitment and activity of cytotoxic T lymphocytes, and inhibit the growth and metastasis of liver cancer cells. Thus, TG100-115 can regulate the transformation of macrophages recruited by sorafenib into M1 phenotype, and play a synergistic anti-tumor effect. The results of the combination of TG100-115 and sorafenib also confirmed the above inference and the tumor inhibition rate reached 63.48%. Increasing the distribution and accumulation of drugs in tumor tissues is the key to ensuring the efficacy of drugs, while the distribution of small molecule drugs often lacks system selectivity. Nanomedicines can increase the selective distribution of drugs, cellular uptake, and reduce toxic side effects. In this study, the prepared micelles showed better antitumor effects in comparison with free drugs solution groups. In comparison, for mice treated with HES-TG100-115-CDM-PEG micelles, the mean tumor weight and inhibition rate were 0.52 ± 0.10 g and 88.70% respectively, which was smaller than free drugs solution group. The reason may be as following: (1) The micelles can be stably present in the blood and utilize the EPR effect of tumor blood vessels to deliver more drugs to the tumor tissue, increasing the tissue selectivity of the drugs and reducing the toxic side effects of the system. (2) The combination of TG100-115 and sorafenib has obvious synergistic anti-tumor effect. Meanwhile, the treatment strategy of killing tumor cells and improving the tumor microenvironment is more conductive to the treatment and prognosis of tumors than traditional specific treatment due to the complexity of the tumor. The construction of micelles ensures the efficacy of the drugs. (3) Drugs can rapidly release in tumor tissue by synthesizing degradable materials and acid-sensitive linkages, thereby ensuring the efficacy of the drugs. Thus, HES-TG100-115-PEG micelles showed satisfactory anti-tumor effect. Meanwhile, it may provide a new direction for the treatment of tumors.

### Evaluation of micelles renal toxicity

**Figure 4.**
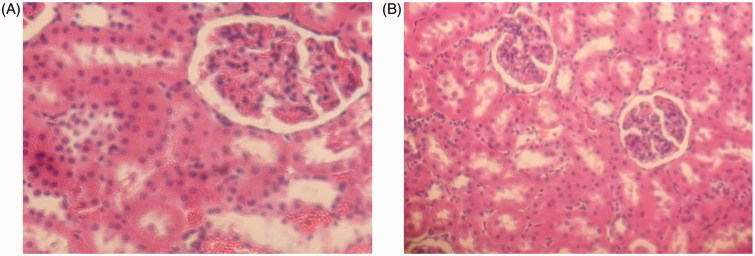
Histology images of rat kidney tissue. (A) 100 mg/mL; (B) 500 mg/mL.

The kidney toxicity of HES-CDM-PEG was evaluated by rats in this paper, and the specific results are shown in Figure 4. It was found that low concentration group did not show significant renal toxicity, including interstitial hyperemia. In contrast, high concentration group showed a certain degree of interstitial hemorrhage in renal pathological sections, but all were not serious. The reason may be as following: on the one hand, PEG is a non-degradable polymer, high concentrations of PEG may cause kidney accumulation and toxicity; on the other hand, HES is not fully degraded by α-amylase and may cause accumulation of HES. However, the amount of HES and PEG is much lower than 100 mg/mL in the process of applying micelles, so no obvious renal toxicity is expected.

## Conclusions

To sum up, sorafenib-loaded liver cancer microenvironment responsiveness HES-TG100-115-CDM-PEG micelles have been prepared and characterized. The particle size of the prepared micelles was about 71 nm, which is an ideal size that promotes the selective distribution of the micelles in tumor tissues utilizing the EPR effect of tumor blood vessels. Meanwhile, the micelles showed good circulation stability and pH-responsive drug release property, which will help reduce the systemic toxicity of the drug and improve the tolerance. The pharmacokinetic results demonstrated that the micelles can significantly alter *in vivo* behavior of TG100-115 and sorafenib, especially for the half-life and bioavailability. Compared with free drugs, micelles exhibited better anti-tumor activity in nude model bearing Hep-3B cell, with lower systemic toxicity, which may indicate good prospect for liver cancer treatment. At the same time, the results also show that it is feasible to combine chemotherapy drugs with tumor microenvironmental regulation drugs for the treatment of tumors. Additionally, the content presented in this article is only a preliminary research, and more in-depth mechanism researches are underway.
